# Monocyte-macrophages modulate intestinal homeostasis in inflammatory bowel disease

**DOI:** 10.1186/s40364-024-00612-x

**Published:** 2024-08-02

**Authors:** Huiying Lu, Zhimin Suo, Jian Lin, Yingzi Cong, Zhanju Liu

**Affiliations:** 1 Department of Gastroenterology, Huaihe Hospital of Henan University, Henan Province, Kaifeng, 475000 China; 2 Center for Inflammatory Bowel Disease Research and Department of Gastroenterology, Shanghai Tenth People’s Hospital of Tongji University, No. 301 Yanchang Road, Shanghai, 200072 China; 3 Division of Gastroenterology and Hepatology, Department of Medicine, Feinberg School of Medicine, Northwestern University, Chicago, IL 60611 USA; 4 Center for Human Immunology, Feinberg School of Medicine, Northwestern University, Chicago, IL 60611 USA

**Keywords:** Macrophage, Inflammatory bowel disease, Intestinal mucosa, Susceptibility genes, Homeostasis, Immunopathology

## Abstract

**Background:**

Monocytes and macrophages play an indispensable role in maintaining intestinal homeostasis and modulating mucosal immune responses in inflammatory bowel disease (IBD). Although numerous studies have described macrophage properties in IBD, the underlying mechanisms whereby the monocyte-macrophage lineage modulates intestinal homeostasis during gut inflammation remain elusive.

**Main body:**

In this review, we decipher the cellular and molecular mechanisms governing the generation of intestinal mucosal macrophages and fill the knowledge gap in understanding the origin, maturation, classification, and functions of mucosal macrophages in intestinal niches, particularly the phagocytosis and bactericidal effects involved in the elimination of cell debris and pathogens. We delineate macrophage-mediated immunoregulation in the context of producing pro-inflammatory and anti-inflammatory cytokines, chemokines, toxic mediators, and macrophage extracellular traps (METs), and participating in the modulation of epithelial cell proliferation, angiogenesis, and fibrosis in the intestine and its accessory tissues. Moreover, we emphasize that the maturation of intestinal macrophages is arrested at immature stage during IBD, and the deficiency of MCPIP1 involves in the process via ATF3-AP1S2 signature. In addition, we confirmed the origin potential of IL-1B^+^ macrophages and defined C1QB^+^ macrophages as mature macrophages. The interaction crosstalk between the intestine and the mesentery has been described in this review, and the expression of mesentery-derived SAA2 is upregulated during IBD, which contributes to immunoregulation of macrophage. Moreover, we also highlight IBD-related susceptibility genes (e.g., RUNX3, IL21R, GTF2I, and LILRB3) associated with the maturation and functions of macrophage, which provide promising therapeutic opportunities for treating human IBD.

**Conclusion:**

In summary, this review provides a comprehensive, comprehensive, in-depth and novel description of the characteristics and functions of macrophages in IBD, and highlights the important role of macrophages in the molecular and cellular process during IBD.

## Introduction

Inflammatory bowel disease (IBD) is a group of chronic relapsing inflammatory diseases that affect the entire gastrointestinal tract, including Crohn’s disease and ulcerative colitis [1, 2]. During gut inflammation, the integrity of the intestinal mucosal barrier is compromised, leading to exposure to commensal microbiota and pathogens [1, 3]. Recent studies have highlighted that the destruction of intestinal mucosal barrier integrity and the disruption of intestinal mucosal immune homeostasis are strongly associated with the dysregulated immune response to commensal microbiota [3–5]. Therefore, it is highly desired to investigate the great significance and complex mechanisms whereby macrophages regulate mucosal homeostasis during IBD.

Intestinal resident macrophages are at the front line of host defense at the mucosal area and form a paramount nexus in maintaining intestinal homeostasis and modulating inflammatory response in gut mucosa [2, 6, 7]. Increasing lines of evidence has indicated that macrophages in the intestine are involved in the generation of cytokines, chemokines, and macrophage extracellular traps (METs), the modulation of immune cell–cell crosstalk, the activation of phagocytosis and bactericidal effects, the response to exogenous antigens and bacteria, and process of epithelial cell proliferation, angiogenesis, tissue repair and fibrosis [6–9]. In line with our recent report [10], perturbation of the intestinal homeostasis contributes to the dramatic changes in the compositions of the intestinal monocyte-macrophage lineages in the pathology of IBD, characterized by the replacement of tissue-resident macrophages by infiltrating proinflammatory monocytes-macrophages [11]. Specifically, excessive or prolonged activation of proinflammatory macrophages results in the impairment of tissue repair and the enhancement of fibrosis, which is associated with an aggravation of surgical risks (e.g., stenosis and intestinal obstruction) [12]. In addition, our recent data have identified that macrophages-derived serum amyloid A2 (SAA2) is positively correlated with the microbial transporters and modulates immune homeostasis in the mesentery, mesenteric lymph nodes and mesenteric adipose tissues [13].

Previous studies have highlighted that copious amounts of IBD susceptibility genes are related to the monocyte-macrophage lineages like nucleotide binding oligomerization domain containing 2 (NOD2), autophagy related 16 like 1 (ATG16L1), C-X3-C motif chemokine receptor 1 (CX3CR1), Janus kinase 2 (JAK2), and signal transducer and activator of transcription 3 (STAT3) [14–17]. The latest breakthrough from our team has illuminated the newly founded genetic characteristics of IBD in East Asian ancestry, including macrophage-related genes like RUNX family transcription factor 3 (RUNX3), IL21R, general transcription factor Iii (GTF2I), and leukocyte immunoglobulin like receptor B3 (LILRB3) [17]. Subsequently, a reduction in pro-inflammatory macrophage polarization ascribed to anti-TNF treatment (e.g., infliximab) and a declination in monocyte accumulation on account of anti-α4β7 integrin therapy (e.g., vedolizumab) lead to clinical remission in IBD [18, 19]. Thus, integrating the definite properties of intestinal monocyte-macrophage lineage under physiological and pathological conditions and translating these findings into future intervention strategies have essential implications for better understanding the pathogenesis and alleviating the clinical manifestations of IBD.

In this review, we discuss the origin and location, the maturation and differentiation, and the function of macrophages in intestinal mucosa sequentially. Importantly, we leverage recently developed technologies, including single-cell RNA-sequencing (scRNA-seq), fate mapping techniques, genome-wide association studies (GWAS), and ImmunoChip, which are instrumental in improving our understanding of the biology and pathology in macrophages. Moreover, we also characterize the features of macrophages during IBD and delineate the relationship between IBD susceptibility gene-driven macrophage activation and potential therapeutics, which concomitantly provide vast connotations for our understanding of macrophage immunoregulation and tissue repair in IBD.

## Origin and location of macrophage in the gut

Macrophages in the intestine contribute to a robust payload of immune regulation, which is markedly distinct from circulating monocytes, and the intestine is the largest reservoir of macrophages in adult tissues [11, 20]. In terms of the anatomical distribution of intestinal macrophages, accumulating lines of evidence have observed that macrophages are strategically localized at the different layers of the entire GI tract, ranging from the lamina propria (LP), which closes proximity to the epithelial monolayer, to the submucosa plexus and muscularis externa [3, 7, 11, 21]. They are also connected to the crypt base within the villi right around lymphoid tissues, the enteric neurons, and blood vessels and are involved in regulating intestinal motility [11, 21, 22]. Interestingly, the number of macrophages changes gradually in the mouse intestine from the proximal to distal ends while this concept is not applicable for macrophages in the human intestine, where they appear to distribute evenly [11].

In the early 1970s, it was described that embryonic precursor macrophages colonizing the tissues in the embryonic stage emanate from either Yolk sac (YS) erythro-myeloid precursors (EMP) or fetal-liver precursors. These lifelong tissue-resident macrophages obtain minimal supplement from adult hematopoietic cells [23, 24]. However, tissue-resident macrophages in the fetal and neonatal intestine, although colonized by embryonic precursors from E8.5 onwards, are reported to have a 3-week half-life and unable to last into adulthood due to their poor proliferative capacity and the exposure to the intestinal microbiota and its metabolites [7, 8, 11, 25, 26]. Therefore, continuous recruitment and replenishment of CCR2-dependent circulating Ly6C^hi^ monocytes, which arise from postnatal hematopoiesis and mature into tolerogenic IL-10^+^ macrophages, are of great importance for the immune homeostasis of intestinal mucosal under physiological and pathological conditions with a unique turnover rate [7, 25, 27]. Intestinal macrophages are distinguished from other immune cells in the gastrointestinal tract with their expression of colony-stimulating factor 1 receptor (CSF1R), CD68, and FcγRI (also known as CD64) [28, 29]. Flow cytometric analysis reveals that the heterogeneous phenotypes of intestinal macrophages differ from circulating Ly6C^hi^ monocytes based on specific surface markers [20]. By combining flow cytometric analysis with principle component analysis, infiltrated monocytes are observed to fail to differentiate into macrophages but remain in an activated status in steady states [30]. A subsequent study has identified a unique genetic signature of intestinal macrophages which differentiate from circulating monocytes with global transcriptomic analysis [31].

Since the twenty-first century, the notion that circulating monocytes have the potential to apply to all adult tissue macrophages has been challenged [23]. The recently developed technologies, including fate mapping techniques and scRNA-seq, have shed some light on understanding the mononuclear phagocyte system, and additional subsets of the monocyte-macrophage population have been deciphered [27, 32, 33]. scRNA-seq enables the differentiation of macrophages from intestinal LP into 4 subpopulations with distinction in their unique localization and functions [34]. Seminal studies have further revealed that several subpopulations of macrophages arising from embryonic precursors in the gut niches maintain locally reflected by their distinct transcriptional profiles and contribute to ontogeny as well as intestinal homeostasis independently of replenishment by circulating Ly6C^hi^ monocytes [23, 33–35]. Additionally, other studies have proposed that most of these tissue-resident macrophages arising from embryonic precursors are constituted by late c-Myb^+^ EMP-derived fetal liver monocytes rather than early EMP-derived YS macrophages [27, 33]. However, an opposite paradigm uncovered that fetal hematopoietic stem cell (HSC) wave, which originates from aorta-gonad-mesonephros (AGM) but not YS EMPs, is the origin of intestinal resident macrophages [36]. In particular, distinct expression of Tim-4 and CD4 could separate intestinal macrophages into three subsets, including macrophages locally maintained (Tim-4^+^CD4^+^), macrophages turnover from circulating monocytes slowly (Tim-4^-^CD4^+^), and macrophages with the high monocyte-replenishment rate (Tim-4^-^CD4^-^) [35]. On the contrary, Tim4 is absent in the intestinal macrophages of human [29]. Moreover, macrophages from the colon LP cells can also be divided into several subsets based on the expression of F4/80 and CD11b. F4/80^hi^ macrophages are considered intestinal resident macrophages, while CD11b^hi^ macrophages are thought of as infiltrating macrophages with replenishment by circulating monocytes [36, 37].

The gradual replenishment and maturation of these hematopoietic stem cell-derived progenitors in the intestinal mucosa as well as the formation of their distinctive surface markers are shaped by the environmental element, including the high commensal burden, and pro-inflammatory and anti-inflammatory signatures [25, 31, 34, 35]. The surrounding environment has a concept that is also known as the macrophage ‘niche’. Signals in the niche involve generating niche-specific phenotypes and functional plasticity of macrophages by activating specific transcriptional profiles within tissue resident macrophages [7]. On the other hand, the death of macrophages in the niche facilitates the recruitment and maturation of monocytes to fill the niches [38]. Previous work has described that the monocyte-macrophages are remarkably downregulated in the colon of germ-free (GF) mice compared with those in conventional control mice. In particular, markedly fewer Ly6C^hi^MHC II^-^ and Ly6C^+^MHC II^+^ cells are observed in the colon of GF mice compared with those in conventional control mice [25]. In addition, the proportion of intestinal macrophages, in particular CD11c^+^CD206^int^CD121b^+^ and CD11c^-^CD206^hi^CD121b^-^ macrophages, is selectively decreased in GF mice compared with that in SPF mice [39]. Moreover, *Enterobacteriaceae*, a specific microbiota, is involved in the recruitment of Ly6C^hi^CCR2^+^ monocytes from the circulation into intestinal mucosa in dextran sulfate sodium salt (DSS)-induced colitis model [40], indicating the critical role of microbiota in modulating the composition of intestinal mucosal macrophages [25, 39]. Evidence has also shown that the generation of tissue-resident macrophages deriving from either HSC-dependent or independent fetal-liver precursors relies on c-Myb activity [33]. The differentiation of intestinal monocytes-macrophages is also modulated by environmental factors like IL-10, TGF-β, colony-stimulating factor 1 (CSF1), and oxysterol [2]. Accordingly, IL-10 is required to suppress the hyperactivation of tissue-resident macrophages in the gut [41]. The IL-10–IL-10R signaling blockage leads to the remodeling of intestinal macrophage phenotypes by restricting the activation of STAT3 [41]. TGF-β, a tolerogenic signal, is released from intestinal epithelia and indirectly induced by gut microbiota, and the TGF-βR1-dependent signaling is involved in the terminal monocyte-macrophage maturation by orchestrating the genetic signature of intestinal macrophages, which is associated with an enhancement of genes including *CX3CR1*, *IL-10*, and *CCDC23* [2, 20, 31]. The mechanisms underlying the TGF-βR1-mediated signaling in regulating the constitution of the intestinal macrophage pool appear to be distinct from those used by IL-10 [31]. CSF1, the primary regulator of the mononuclear phagocyte lineage, participates in the proliferation of intestinal macrophage maturation. Consistently, deficiency of CSF1 in mice leads to the suppression of macrophage differentiation [42]. Silico trajectory analysis uncovers that the localization and function of intestinal macrophages are modulated by extravasated monocytes and key transcription factors [8, 29]. Depletion of these tissue-resident macrophages leads to the Csf-1R/Csf-2 signaling-dependent recovery of intestinal macrophages and reduction in intestinal motility [23, 34]. As for oxysterol levels, it seems that the enhancement of oxysterol subsequently recruits GPR183^+^ inflammatory circulating monocyte-derived cells into inflamed intestine [38].

Taken together, cumulative data have confirmed that embryonic original tissue-resident macrophages as well as peripheral-derived macrophages co-existed in the intestinal mucosa, and their compositions and distribution are tremendously dependent on signatures in the niches. 

## Macrophage maturation

### The process of macrophage maturation

As described above, intestinal-resident macrophages lack the ability of self-sustainment, and the continuous recruitment and maturation of circulating monocytes are required to fill the intestinal niches and maintain the homeostasis of intestinal mucosal immunity in a steady state [11, 38, 43]. This process is triggered by the release of pro-inflammatory mediators, cytokines, and chemokines (e.g., CCL2, CCL8) [6]. After entering into the LP of intestinal mucosa, CCR2^+^Ly6C^+^ monocytes initiate a series well-defined process of maturation, which acquire major histocompatibility complex II (MHC II) firstly, followed by losing the expression of Ly6C and CCR2, and the enhancement of F4/80, CD64, CX3CR1 and CD206 in mice, or the enhancement of CD64, CD68, CD206 and CD163 in humans [2, 6, 7, 11, 25, 28, 43]. By using flow cytometry, the process of the monocyte-to-macrophage maturation continuum is identified in both mouse and human intestines [24, 31]. Gating strategy is exemplified: Monocytes and macrophages filled the LP of the intestinal mucosa are gated according to the high expression of CD45, CD11b, and CD64 [28]. After that, these gated cells are divided into four populations according to their maturation status. Newly infiltrated monocytes are defined as population 1 (P1) and characterized by a unique phenotype that expresses a high level of Ly6C, but the expression of MHC‧II is absent (namely, Ly6C^hi^MHC‧II^+^). Subsequently, monocytes that gradually acquire MHC‧II expression are reckoned as immature macrophages and named population 2 (P2, Ly6C^+^ MHC‧II^+^) to “bridge” monocytes and mature macrophages. Finally, immature macrophages lose their expression of Ly6C and then obtain the characteristics of mature macrophages, namely population 3 (P3, Ly6C^-^MHC II^+^CX3CR1^int^). Mature macrophages fill the intestinal niche and are classified as population 4 (P4, Ly6^-^CMHC II^+^CX3CR1^hi^) [3, 24, 28, 31, 43]. Given that the P3 and P4 cells are only distinguished by differential expression of CX3CR1, they are considered as a single population 3/4 (P3/P4, Ly6C^-^MHC II^+^CD64^+^) in mice [28]. This entire maturation process is called monocyte-waterfall, which usually takes almost 5–7 days [11, 24]. In addition, flow cytometry also distinguishes macrophages from DCs in the LP of intestinal mucosa from both mice and humans through the expression of CD64 [28]. These mature macrophages from the LP of mouse intestinal mucosa develop characteristic functions like the enrichment of receptors associated with phagocytic and bactericidal activity, including TIM4, CD36, and αvβ5 integrin, IL-10 production, apoptotic cell elimination, as well as the tolerance of TLR stimulation [3, 11, 31, 43–45].

The application of unbiased scRNA-seq reveals the differences in transcriptomes among monocytes, immature macrophages, and macrophages from intestinal mucosa [8, 31, 46, 47]. Several subsets of mRNA transcripts involved in an extravasation process and TLR and pro-inflammatory associated processes are significantly downregulated during the maturation process from the newly infiltrated monocytes (P1) to mature macrophages (P4) in the LP of intestinal mucosa, including *Ly6c1*, *Itgb7*, *Sell*, *Itga1*, *Ccr2*, *Gpr35*, *Myd88*, *Irak3*, *Trem1*, and *Il6* [31]. In contrast, mRNA transcripts associated with phagocytosis (*Mertk*, *Mrc1* (CD206), *Cd36*, *Gas6*, *Axl*, *Itgav*, *Itgb5*, *Cd9*, *Cd81*, *C1qa-c*, *Klf2*, *Stab1*), metalloproteinases (*Mmp2*, *Mmp9*, *Mmp12*, *Mmp13*), TGFβR signaling (*Tgfbr1*, *Tgfbr2*, *Smad7*, *Serpine1*), and monocyte chemoattractants (*Ccl7* and *Ccl8*) are upregulated during maturation process from the P1 to P4 in the LP of intestinal mucosa [8, 31, 46]. These observations emphasize that mature macrophages are more involved in phagocytosis, anti-inflammation, and tissue repair and are consistent with previous studies described above. In addition, the mRNA transcriptions of *S100A8*, *S100A9*, *CD209*, *CD163*, *RXRA*, *MPO*, *TRAF3IP3*, *PFKP*, *FCN1*, and *VCAN* are restricted, while the mRNA transcriptions of *CD169*, *MHC II*, *ACP5*, *C1Q*, *ICA1*, *GOPC*, *STOML1*, *KIFAP3*, and *KIF3C* are enhanced during the maturation from the newly infiltrated monocytes to mature macrophages in the LP of intestinal mucosa [8, 46, 47].

### Regulation of macrophage maturation

The maturation of monocytes to macrophages mainly depends on the tolerogenic environment in intestinal niches and the integrity of the intestinal epithelial barrier [6, 11, 24, 38, 48]. For instance, commensal microbiota and its metabolites in the intestinal mucosa orchestrate the recruitment of circulating monocytes and constantly fine-tune the fulfillment of mature macrophages in intestinal niches under both physiological and pathological conditions [2, 28, 49]. Chemokines and their receptors also participate in the process of the monocyte-macrophage maturation. CX3CR1 is known as a TGF-β-dependent chemokine receptor, bound by CX3CL1, thus indicating the maturation of macrophages [8, 31]. The niche environment in the gut instructs an upregulation of CX3CR1 in monocytes-macrophages since macrophages in other tissues lack the expression of CX3CR1 [11]. Thereafter, CX3CR1^hi^ macrophages in the gut secrete high levels of CCL2 and CCL7 and consequently fine-tune their replenishment [11, 50, 51]. Published data have also revealed that the monocyte-macrophage maturation is largely dependent on the CSF1/CSF1R signaling, and multiple growth factors like flt3L, CSF1R, CSF1, and CSF2 are also involved [8, 11, 24, 40, 52–54]. It should be noted that CSF1 functions in a concentration-dependent manner since a high level of CSF1 facilitates macrophage proliferation while a low level of CSF1 participates in macrophage survival [7, 55–57]. In contrast, anti-CSF-1R monoclonal antibody treatment significantly interferes with the monocyte-macrophage maturation but does not influence the infiltration of pro-inflammatory monocytes [57, 58]. In the local environment of the gut, a large amount of IL-10 and IFN-γ secreted by Treg cells, and epithelium-derived mediators, including CX3CL1, IL-33, and IL-25 also involved in the modulation of the monocyte-macrophage maturation [6, 8, 59–61]. IL-10 has been confirmed to upregulate the expression of CD206 and CD163, and guarantee the generation of MHC II, leading to the monocyte-macrophage maturation [11]. Interestingly, the generation of IL-10 is regulated by macrophages, which contribute to a potent payload in maintaining the immunity homeostasis of intestinal niches [6]. Cumulative lines of evidence have demonstrated that several transcription factors are involved in the development of macrophages in the colon, exemplified by triggering receptors expressed on myeloid cells 1 (TREM1), which acts as an amplifier of pro-inflammatory responses [8, 62]. Additionally, our recent data have uncovered that monocyte chemotactic protein-1-induced protein 1 (MCPIP1) orchestrates monocyte to macrophage maturation in the intestine via a activating transcription factor 3 (ATF3)-adaptor related protein complex 1 subunit sigma 2 (AP1S2)-dependent manner and contributes to the restraint of mucosal inflammation [10]. Evidence also verifies that zinc finger E-box-binding homeobox 2 (ZEB2) is involved in maintaining the tissue-specific identities of macrophages in the intestine [8, 63]. In addition, the circadian clock also participates in the process of macrophage maturation [38]. Collectively, intestinal niches exert a crucial influence on shaping the fate of the monocyte-macrophage lineage.

### Macrophage maturation during colitis

Evidence has shown that monocyte maturation to macrophages is arrested at an immature state (P2, Ly-6C^int^ MHC II^+^CD64^+^) during inflammation, including IBD [3, 10, 24, 28, 31, 43, 50, 60, 64]. Circulating monocytes (Ly6C^hi^MHC II^-^, equivalent to CD14^hi^CD11c^hi^CCR2^+^CX3CR1^+^ monocytes in humans) infiltrate into intestinal mucosa massively, while the process of its maturation into mature macrophages (Ly6C^-^MHC II^+^CD64^+^, equivalent to CD14^hi^CD11c^-^CCR2^-^CX3CR1^-^ macrophages in humans) is disrupted compared with that at steady state, leading to the enrichment of immature macrophages (Ly6C^+^MHC II^+^CD64^+^, equivalent to CD14^hi^CD11cCCR2^-^CX3CR1^+^ immature macrophages in humans) and the exhaustion of mature macrophages [6, 10, 24, 28]. These immature macrophages are different from ‘classical macrophages’ and highly responsive to the TLR signaling [24, 43, 65], contributing to an upregulation of pro-inflammatory mediators (TNF-⍺, IL-1β, IL-6, IL-12, IL-23, iNOS, and OSM) and downregulation of IL-10, leading to monocyte infiltration, tissue damage and function deterioration [6, 24, 28, 38, 66]. Taken together, these inflammation-associated signals further skew the process of the monocyte-macrophage maturation toward a disordered status [38].

## Macrophage heterogeneity

Previous studies have roughly divided macrophages into pro-inflammatory macrophages (M1) and anti-inflammatory macrophages (M2). However, with the advanced technology, especially scRNA-seq, macrophages are now further divided into several subpopulations based on distinct classification dimensions **(**Table 1**)**.

**Table 1:** Macrophage heterogeneity in the intestine

Population	Markers	Characteristics	References
M1	inos, IL-1β, IL-6, CCL5, CXCL9, MMP9	1) Activated by LPS and/or IFN-	[67–71]
		2) Metabolized by glycolysis	
		3) Deregulate tight junction proteins	
		4) Increase apoptosis of epithelial cell	
		5) Disrupt epithelial barrier	
M2	CD206, CD163, CCL17, IRF4, ARG-1, Retnla, Chi3l3	1) Activated by IL-4, or IL-13	
		2) Repair damaged tissue	
		3) Regulated IL-10 and TGF-β1 producing Treg cells	
		4) Produce IL-10 and ARG-1	
		5) Exacerbate pathological fibrosis	
Tim-4^+^CD4^+^ macrophages	Tim-4^+^CD4^+^	Rarely replaced by infiltrated monocytes	[35]
Tim-4^-^CD4^+^ macrophages	Tim-4^-^CD4^+^	Slowly replaced by infiltrated monocytes	
Tim-4^-^CD4^-^ macrophages	Tim-4^-^CD4^−^	High replenishment rate from infiltrated monocyte	
Mϕ1	CD11b^hi^ CD64^+^ MerTK^+^	Similar to peripheral blood monocytes phenotypically	[47]
	CD163^+^ CD115^+^ CX3CR1^+^		
	CD206^-^ CD1c^-^ CD103^−^		
	CCR2^+^ calprotectin^+^		
Mϕ2	CD11b^+^ CD64^+^ MerTK^+^		
	CD163^med^ CD115^+^		
	CX3CR1^med^ CD206^+^ CD1c^+^		
	CD103^+^ CCR2^+^ calprotectin^+^		
Mϕ3	CD11b^-^ CD64^low^ MerTK^+^	Similar to mature macrophages phenotypically	
	CD163^+^ CD115^+^ CX3CR1^-^		
	CD206^+^ CD1c^−^ CD103		
	^-^ CCR2^-^ calprotectin^-^		
Mϕ4	CD11b^+^ CD64^low^ MerTK^+^		
	CD163^+^ CD115^low^ CX3CR1^low^		
	CD206^+^ CD1c^-^		
	CD103^-^CCR2^-^ calprotectin^-^		
monocyte-like cells	CD11c^high^ CCR2^+^	1) Share characteristics with circulating CD14^+^ monocytes	[90]
	CX3CR1^+^	2) Increased in the inflamed intestinal mucosa from IBD patients	
		3) Produce IL-1β	
macrophage-like cells	CD11c^-^ CCR2^-^ CX3CR1^-^	1) macrophage-like tissue resident counterparts	
		2) Produce IL-10	

### Pro- and anti-inflammatory macrophages

Mature macrophages can polarize into ‘classically activated’/pro-inflammatory macrophages (M1) or ‘alternatively activated’/anti-inflammatory macrophages (M2), characterized by surface receptor expression, mediator secretion, and cell functions [2, 67–70]. With the activation of LPS (TLR ligand) and IFN-ℽ, macrophages are polarized into M1-like macrophages dependent on the TLR4 activation and NF-ĸB facilitation, which are metabolized by glycolysis and identified through the expression of inos (NOS2), IL-1β, IL-6, CCL5, CXCL9, and MMP9 [67–71]. These pro-inflammatory cytokines and inos lead to epithelial barrier disruption and tissue damage due to the deregulation of tight junction proteins and upregulation of epithelial cell apoptosis, predisposing to IBD [72]. The accumulation of iron, cholesterol, and other sterile inflammatory signals in pro-inflammatory macrophages enhances TNF-α production and further impairs wound-healing [57, 73].

Additionally, macrophages stimulated with IL-4 or IL-13 can polarize into M2-like macrophages, committing to oxidative phosphorylation (OXPHOS) and expression of CD206, CD163, CCL17, IRF4, ARG-1, Retnla and Chi3l3 [2, 67, 71]. These M2-like macrophages are involved in tissue repair during inflammation directly or through the regulation of IL-10- and TGF-β1-producing Treg cells [57, 74]. Interestingly, endotoxin-tolerant macrophages also produce IL-10 and arginase 1, participating in tissue repair and immune regulation [75]. However, overactivation of M2-like macrophages has been found to exacerbate pathological fibrosis [57, 76]. Previous studies have demonstrated that cytokines like IL-6, IL-10, and IL-21 facilitate anti-inflammatory function and M2 polarization of macrophages by enhancing IL-4 receptor expression, as characterized by the upregulation of tissue repair and regeneration [40, 57, 77, 78]. In contrast, TNF-α production interferes with M2 polarization of macrophages and thus counteracts tissue repair [57, 73].

Evidence has uncovered that several biological processes are also involved in regulating macrophage polarization. Methyltransferase Setdb2 has been found to facilitate macrophage polarization from inflammatory phenotype to a reparative status by trimethylating histone 3 at NF-κB binding sites on inflammatory cytokine gene promoters [79]. Suppression of DNA methylation through the deletion of DNA methyltransferase 1 (DNMT1) results in the enhancement of alternatively activated macrophages [80]. PGE2 has been demonstrated to downregulate the secretion of pro-inflammatory cytokines and promote the activation of M2 polarization, which is modulated by the cyclic AMP (cAMP)-protein kinase A pathway, cAMP-responsive element-binding protein and Kruppel-like factor 4 [81]. However, other studies suggest that the proliferation of M2-like polarization of macrophages and the expression of M2-related marker genes is suppressed by PGE2 [82]. Besides, PGE2 inhibits oxidative phosphorylation activity via the downregulation of tricarboxylic acid-cycle intermediates [82]. Moreover, macrophage polarization is also modulated by a variety of micro-RNAs (miRs). miR-720 [83], miR-223 [84], miR-127 [85], and miR-155 [86] have been found to contribute to the M1 polarization by targeting GATA3, C/EBPβ, and BCL6, respectively, while miR-378–3p [87], miR-511–3p [88], and miR-146a [87] facilitate the M2 polarization by targeting PI3K/AKT1, ROCK2, Notch1, and PPAR-ℽ, respectively [70].

### Unclassical classification criterion

In addition to the classification criterion that identifies macrophages into M1-like and M2-like macrophages, emerging evidence distinguishes macrophages based on the expression of Tim-4 and CD4 [35]. Tim-4, highly expressed by Kupffer cells, acts as an upstream in the apoptotic cell-eradication process [35, 89]. Tim-4^+^CD4^+^ macrophages in intestinal mucosa are observed to be maintained locally and rarely replaced by circulating monocytes. In contrast, accumulated monocytes replenish intestinal mucosa via the replacement of Tim-4^-^CD4^-^ macrophages. Tim-4^-^CD4^+^ macrophages ‘bridge’ these two phenotypes and can be slowly interchanged by circulating monocytes [35].

Synthesized analysis of surface markers has manifested the heterogeneity of macrophages and classified human intestinal macrophages into several subsets. Bujko et al. have identified small intestinal macrophages into four subsets according to surface markers, including CD14, CD11b, HLA-DR, and CD11c with the utilization of flow cytometric analysis [6, 47], namely Mϕ1 (CD11b^hi^CD64^+^MerTK^+^CD163^+^CD115^+^CX3CR1^+^CD206^-^CD1c^-^CD103^-^CCR2^+^calprotectin^+^), Mϕ2 (CD11b^+^CD64^+^MerTK^+^CD163^med^CD115^+^CX3CR1^med^CD206^+^CD1c^+^CD103^+^CCR2^+^ calprotectin^+^), Mϕ3 (CD11b^-^CD64^low^MerTK^+^CD163^+^CD115^+^CX3CR1^-^CD206^+^CD1c^-^CD103^-^CCR2^-^calprotectin^-^), and Mϕ4 (CD11b^+^CD64^low^MerTK^+^CD163^+^CD115^low^CX3CR1^low^CD206^+^CD1c^-^CD103^-^CCR2^-^calprotectin^-^). Among them, Mϕ1 and Mϕ2 equip the comparable characteristics as circulating monocytes, while Mϕ3 and Mϕ4 share the same features of mature macrophages [47]. In addition, the locations of these populations are distinct from each other. Mϕ3 is predominantly located in the intestinal villus and forms a network in the LP, whereas Mϕ4 is mainly in the submucosa [47].

Additionally, human intestinal macrophages can also be phenotyped into 2 subsets based on the expression of CD11c, CCR2, and CX3CR1 [90]. Macrophages are identified as CD45^+^HLA-DR^+^CD14^+^CD64^+^ cells, and the subsets are divided into monocyte-like cells (CD11c^hi^CCR2^+^CX3CR1^+^), macrophage-like tissue-resident cells (CD11c^-^CCR2^-^CX3CR1^-^). LPS triggers the expression of IL-1β in monocyte-like cells but suppresses the expression of IL-10 in macrophage-like cells. It should be noted that the enrichment of monocyte-like cells in intestinal mucosa may contribute to the retention of inflammation in IBD patients, while macrophage-like tissue-resident cells are generated during resolution [90].

With the assistance of scRNA-seq, our recent study has unraveled a proinflammatory population (Ccr2^+^Il-1β^+^Tlr2^+^Cx3cr1^-^Cd163^-^Mrc1^-^Ly6c^+^) of the monocyte/macrophage lineage from LP CD11b^+^ cells. During intestinal inflammation in *Mcpip1*^∆Mye^ mice, the maturation from monocytes to macrophages is predominantly arrested, characterized by the enrichment of proinflammatory monocytes subset (Ccr2^+^Il-1β^+^Tlr2^+^Cx3cr1^-^Cd163^-^Mrc1^-^Ly6c^+^), leading to the aggravation of gut inflammation via an Atf3-Ap1s2 axis-dependent manner [10]. After that, a subset of monocyte-macrophage lineage equipped with unique marker genes (e.g., *Cx3cr1*, *Cd163*, *Mrc1*) is identified as mature macrophages, while newly recruited monocytes are identified according to their unique marker genes (e.g., *Ccr2*, *Il-1β*, *Tlr2*, *Ly6c*).

The classification criterion shown above is far from exhaustion, and we believe that the analysis of macrophage phenotypes will continue to be improved with advanced technologies (e.g., scRNA-Seq) in the future.

## The functions of macrophages

The monocyte-macrophage lineage in intestinal LP is indispensable in maintaining intestinal mucosal homoeostasis [6, 7, 11, 24, 35]. Macrophages located snugly under the epithelial layer possess high phagocytic and bactericidal activity and can capture and eliminate pathogenic bacteria that penetrate the lining of the gut [2, 7, 11, 20, 41, 91–93]. Specifically, after being stimulated by pathogenic bacteria or its derivates, hyporesponsive macrophages generate transepithelial dendrites, which can capture *Salmonell*a organisms as well as soluble antigens in the lumen [2, 11, 94, 95]. Accordingly, the antigens are then presented to neighboring migratory dendritic cells, thus contributing to subsequent immunomodulation [2]. Besides pathogenic microorganisms, macrophages can also eliminate apoptotic or senescent cells and cell debris, contributing to host defense of intestinal mucosal homoeostasis [2, 11, 31, 35, 57, 91, 92, 96]. In contrast, eradication of mature macrophage leads to the augmentation of intestinal permeability and epithelial cell death, thus driving the aggravation of intestinal inflammation [6, 97].

Increasing lines of evidence have shown that macrophages produce several types of pro-inflammatory or anti-inflammatory cytokines, chemokines, TLRs, METs, and lipid mediators, which regulate intestinal immune homeostasis [2, 3, 11, 40]. Of note, IL-10 is the most critical cytokine responsible for the modulation of the responses of macrophages to pattern recognition receptor triggering as well as the maintenance of the amplification of FoxP3^+^ Treg cells and ILC3 in the intestinal mucosa [3, 34, 41, 78, 93, 98, 99]. Macrophage-derived IL-1β is also involved in the survival of IL-17-producing CD4^+^ T cells and CSF2 secretion by ILC3 [3, 100, 101]. Moreover, macrophages are one of the primary sources of chemokines, including CCL2, CCL8, CXCL1, and CXCL2, leading to the recruitment of neutrophils, monocytes, and T cells [3, 31, 50]. Macrophages are also required to maximize the bactericidal activity of neutrophils by facilitating suicidal NETosis, a process through which neutrophils form and release neutrophil extracellular traps (NETs) to capture and eliminate bacteria. Simultaneously, NETs promote the phagocytosis and antibacterial activity of macrophages synergistically [102]. Similar to the NETs, METs, composed of cellular DNA, citrullinated histone H3, and MPO, are released from macrophages to capture, immobilize, and kill microorganisms [103, 104]. METs have been clarified to be involved in several pathological processes, including rhabdomyolysis-induced acute kidney injury, iron overload-related liver ischemia/reperfusion injury, and the phagocytosis of *Candida albicans* [103, 105]. However, the role of METs in orchestrating intestinal mucosa immunity has hitherto not been elucidated. Besides, a full range of TLRs is generated by intestinal tissue-resident macrophages [43] and responsible for the downregulation of several adapter molecules, including CD14, MyD88, TRAF-6, MD2, TRIF, and IRAK1 [20, 50], leading to the hyporesponsiveness of these macrophages. Concomitantly, TLR signaling and NF-ĸB activation are suppressed by IRAK-M and IkBNS [50], which are overexpressed in intestinal-resident macrophages [11]. It should be noticed that macrophages also interfere with cell metabolism, facilitate apoptosis, deteriorate ischemic injury through producing reactive oxygen species (ROS) and toxic mediators, and subsequently impede mucosa healing [40].

Macrophages in intestinal LP have also been revealed to be involved in fundamental physiological processes, including maintaining intestinal barrier integrity, proliferation of epithelial cells, angiogenesis, and tissue repair. Through generating mediators like hepatocyte growth factor (HGF) [106], PGE2 [64, 107, 108], WNT ligands [109, 110], and metalloproteinases [31] as well as signaling pathways including NOX1 signaling activated by annexin A1 [111, 112] and CREB-WISP1 signaling activated by IL-10 [113], intestinal macrophages contribute to the renewal of intestinal stem cells in intestinal crypts, proliferation of epithelial progenitors, and promotion of arteriogenesis, leading to the preservation integrity of the mucosa and recovery of ischemic tissue with minimal collateral damage [2, 3, 6, 11, 29, 31, 35, 40, 57, 107]. Besides, MyD88 signaling secreted from myeloid cells also drives intestinal epithelial repair [11, 114]. Interestingly, GeneChip analysis combined with immunostaining and electron microscopy has pointed out that macrophages can connect to colonic epithelial stem cells directly near the crypt base and thus induce epithelial proliferation [7, 107]. Previous studies have confirmed that macrophages synthesize polyamines via a mTORC1-arginase-1-dependent manner that is being taken up by the epithelial cells, contributing to the triggering of metabolic reprogramming and the enhancement of proliferation at a steady or inflammatory state [115]. In addition to epithelial cells, intestinal macrophages also regulate Paneth and goblet cell generation, since macrophage depletion by anti-CSF1R treatment interferes with the differentiation of LGR5^+^ intestinal stem cells into intestinal epithelial cells, Paneth cells and goblet cells [2, 6, 116]. Additionally, deficiency of intestinal macrophages leads to the depletion of VE^-^cadherin^+^ blood vessels and deterioration of the submucosal vascular network [7, 34, 40, 117, 118].

Emerging evidence demonstrated a specialized function of resident macrophages of the muscularis externa (MMϕ) in the enteric nervous system (ENS). ENS, composed of the submucous plexus and the myenteric plexus, controls different intestine processes independently of the brain or spinal cord [6]. MMϕ has been demonstrated to play an indispensable role in meliorating the nascent ENS by pruning synapses and phagocytosing enteric neurons. MMϕ deficiency causes a caspase3-dependent neuronal loss and thus contributes to peristalsis alteration, intestinal secretion downregulation, and intestinal transit abnormity [6, 34, 119]. However, the regulation of intestinal contractility and motility is a consequence of the paracrine release of PGE2 from intestinal macrophages in a neuron-independent manner [120]. In addition, MMϕ are feedback-regulated by the ENS. Contacting with ENS and ENS-derived CSF1 or TGF-β maintains MMϕ homeostasis and facilitates its differentiation into a neurosupportive, long-lived and self-maintained phenotype, contributing to the formation of ENS and maintenance of intestinal transit [6, 21, 34, 119]. Swift activation of extrinsic sympathetic neurons in cooperation with norepinephrine signaling to β2 adrenergic receptors on MMϕ enhances tissue-protective programs [21].

Apart from the data mentioned above, intestinal macrophages exert an indispensable role in intestinal fibrosis [6, 15, 40, 57, 121, 122]. Matrix metalloproteinases (MMPs) derived from macrophages are involved in the regulation of fibrin and collagen turnover through the degradation of extracellular matrix (ECM) proteins [57]. Macrophages generate mediators including TGF-β1, IL-36, platelet-derived growth factor (PDGF), insulin-like growth factor 1 (IGF-1), and connective tissue growth factor (CTGF) to directly activate fibroblasts, leading to the development of fibrosis and facilitation of wound healing [1, 15, 40, 57, 121–124]. In contrast, macrophages also participate in the inhibition of fibrosis directly via the production of IL-10, RELMα, and ARG1 [125–127] and indirectly through the suppression of CD4^+^ T cell proliferation and fibroblast activation [40, 121]. However, the dysregulated immune response of intestinal macrophages leads to inefficient tissue wound-healing in the intestinal mucosa, contributing to chronic tissue injuries or chronic inflammation characterized by an infiltration of immune cells and pathological fibrosis or scarring. This aberrant repair culminates with organ failure and death [40, 57, 76]. Consequently, the accumulation, activation, and elimination of monocytes and macrophages are tightly regulated during gut inflammation.

## Macrophages during gut inflammation

**Fig. 1:**
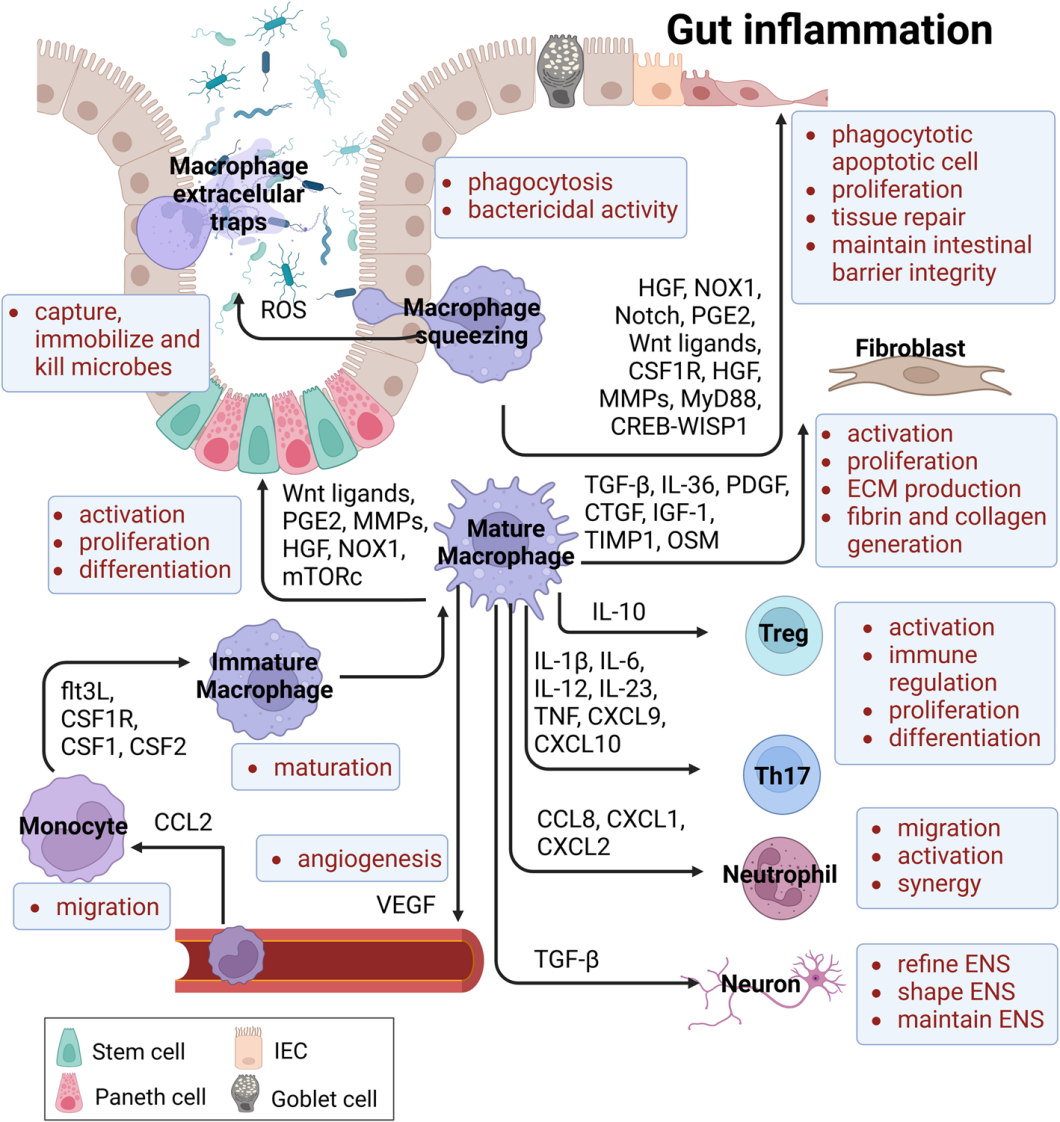
Potential roles of macrophages in intestinal mucosa. The infiltration of circulating monocytes is triggered by a CCL2-CCR2 axis, which consistently maturate into macrophages to modulate intestinal immunity and homeostasis. Macrophages involve in phagocytosis and bactericidal effects, and response to exogenous antigens and microbiotas. Macrophage extracellular traps (METs) are released to capture, immobilize and kill microorganisms. In addition, macrophages upregulate the production of cytokines (e.g., IL-1b, IL-6, IL-10, IL-12, IL-23, TGF-b, and TNF-a) and chemokines (e.g., CCL8, CXCL1, CXCL2, CXCL9, and CXCL10), resulting in the regulation of proliferation, differentiation and immune response of neutrophils and T cells. Additionally, macrophages also contribute to tissue repair with the enhancement of epithelial cell proliferation, angiogenesis and fibrosis

Intestinal pathological encounters, including IBD, predispose to a massive infiltration of CD14^+^ monocytes (equivalent as Ly6C^hi^ monocytes in mice) through the CCL3-CCR1 chemokine axis, which differentiate into macrophages to regulate intestinal immune homeostasis and resolve mucosal wound or inflammation [3, 8, 11, 26, 29, 35, 128–130]. The dysregulation of intestinal macrophages results in intolerance to commensal bacteria and food antigens, and concomitantly contributes to chronic pathological disorders in the gut, including IBD [2, 24, 35, 43, 131, 132]. Here, we introduce the divergent functions of the monocyte-macrophage lineage during IBD **(**Fig. 1**, **graphical abstract).

### The maturation of macrophages during IBD

Evidence has shown that monocyte maturation to macrophages is arrested at P2 stage (immature macrophage, P2, Ly6C^+^MHC II^+^) during gut inflammation, leading to the enhancement of proinflammatory monocytes (P1, Ly6C^hi^MHC II^-^) and immature macrophage (P2, Ly6C^+^MHC II^+^), but exhaust mature macrophages (P3/P4, Ly6C^-^MHC II^+^CD64^+^) [10, 28, 30, 43]. During IBD, infiltrated monocytes and immature macrophages in inflamed mucosa stay responsive to TLRs, contributing to an upregulation of pro-inflammatory mediators (e.g., IL-1β, IL-6, IL-12, TNF-α, iNOS) and bolster respiratory burst activity, and downregulation of IL-10, leading to monocyte infiltration, tissue damage and function deterioration [11, 24, 28, 40, 45, 50, 133, 134]. Mechanically, these pathological processes contribute to the exacerbation of intestinal immune inflammation. Relevant mediators and immune cells in intestinal niches are involved in regulating the monocyte-macrophage maturation process under inflammatory conditions. Since the infiltration and development of monocytes are strictly dependent on CCR2 expression, the enhancement of myeloid cell-derived CCR2 expression in intestinal niches interacts with their ligand CCL2 during inflammation to induce the recruitment of monocytes and immature macrophages and fills the empty niche [28]. Additionally, an upregulation of macrophage maturation in the intestine has been demonstrated after neutralizing with an anti-granulocyte–macrophage colony-stimulating factor (GM-CSF) antibody [135]. In line with this, an IFNℽ-STAT1 axis is required for the maturation of monocytes in the intestine of DSS-challenged mice by triggering histone acetylation at the promoter regions of the Tnf and Nos2 loci [61]. According to our recent study, MCPIP1 deficiency enhances the infiltration of circulating monocytes and restricts monocyte-macrophage maturation via the ATF3-AP1S2 pathway in the intestinal mucosa, leading to the deterioration of intestinal mucosal inflammation upon DSS insult [10]. Synchronously, depletion of innate lymphoid cells (ILC) has been found to restrain the maturation of intestinal monocyte-macrophage lineage, but the number of monocytes remains unchanged in the DSS-induced colitis model [135]. Except for the proinflammatory mediators and immune cells, microbiota and its metabolites in the intestinal niches also participate in the recruitment and maturation of monocyte-macrophage lineage during inflammation [49]. Nevertheless, the retardation of macrophage maturation during intestinal inflammation causes the attenuation of bactericidal and phagocytic ability [2, 136, 137]. In DSS-induced murine colitis model, a transient loss of resident macrophages is uncovered, resulting in an empty macrophage niche in inflammatory intestinal mucosa [138, 139], which is partly ascribed to the affection of sulfated polysaccharides [139]. In addition, the elimination of microbiota in the intestinal downregulates replenishing the “emptied” niches during gut inflammation, suggesting a commensal-dependent refilling manner in the niche [49].

### Phenotypic changes of macrophages during IBD

**Fig. 2:**
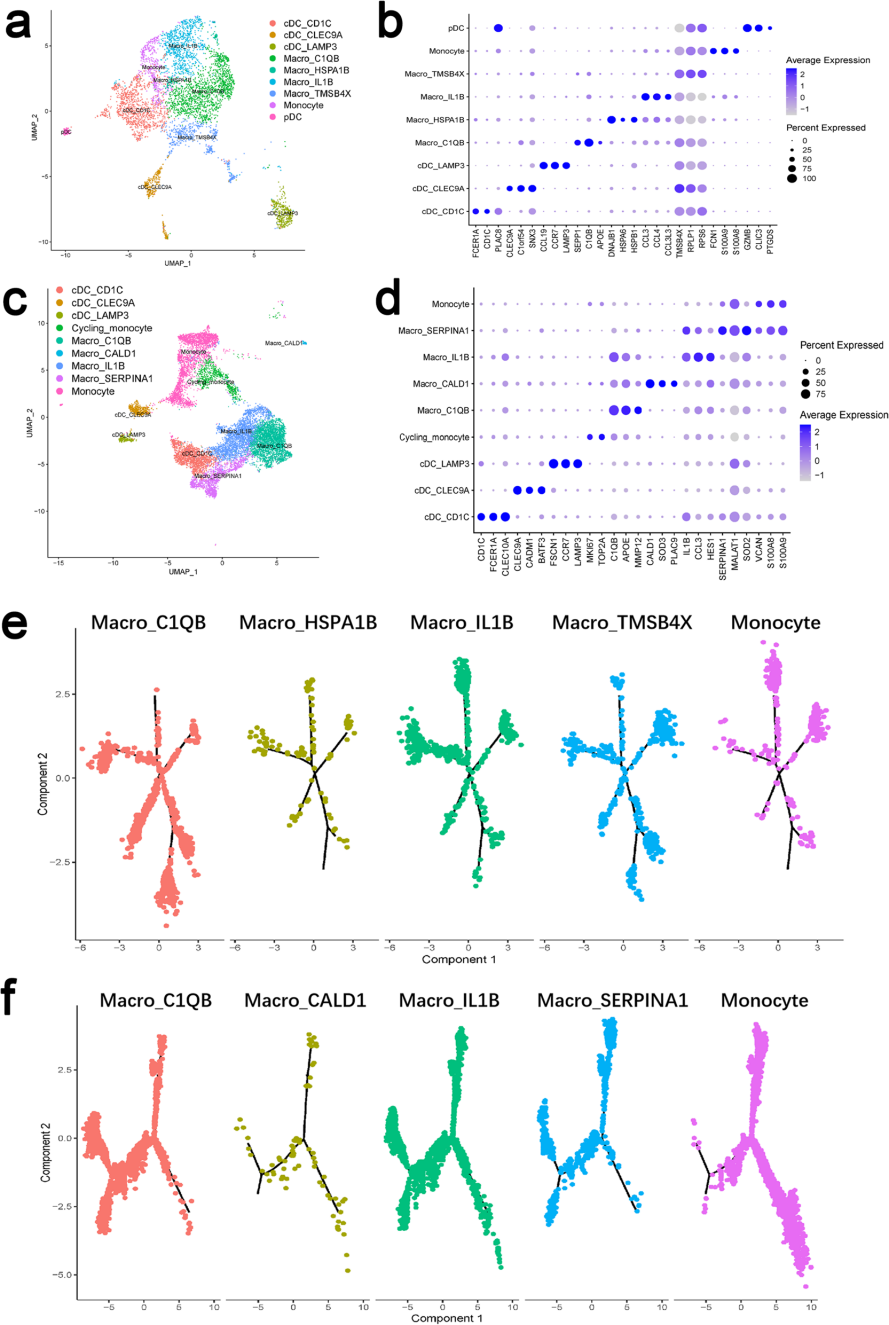
Identification and characteristics of the monocyte-macrophage lineage in intestinal mucosa from IBD patients. (A, C) UMAP plots defines 8 clusters in CD (A) and UC (C) patients, respectively. (B, D) Dot plots showing expression levels of selected signature genes of the monocyte-macrophage lineage subsets in intestinal mucosa from CD (B) and UC (D) patients. Dot size indicates fraction of expressing cells, colored based on the relative expression of specific gene. (E, F) The developmental trajectory of the monocyte-macrophage lineage subsets in CD patients (E) and UC patients (F) inferred by Monocle2

To further classify intestinal macrophages from IBD patients, we retrieved the publicly available scRNA-seq data, including 11 samples from Crohn's disease patients [128] and 18 samples from ulcerative colitis patients [129]. We painted the landscapes of monocyte-macrophages and identified 5 distinct subsets including monocytes, C1QB^+^ macrophages, HSPA1B^+^ macrophages, IL-1B^+^ macrophages, and TMSB4X + macrophages in the intestinal mucosa from Crohn’s disease patients (Fig. 2a). Notably, TMSB4X, RPLP1, and RPS6 are found to be highly expressed in both C1QB^+^ macrophages and TMSB4X^+^ macrophages from Crohn’s disease patients, while CCL3, CCL4 and CCL3L3 are highly expressed in both IL-1B^+^ macrophages and HSPA1B^+^ macrophages, showing a similar convergence in biological characteristics and functions between these two subpopulations (Fig. 2b). Moreover, we also portrayed 5 subpopulations of monocytes, C1QB^+^ macrophages, CALD1^+^ macrophages, IL-1B^+^ macrophages, and SERPINA1^+^ macrophages in the intestinal mucosa from ulcerative colitis patients (Fig. 2c). Interestingly, APOE, C1QB, and matrix metalloproteinase 12 (MMP12) are highly expressed in C1QB^+^ macrophages, IL-1B^+^ macrophages and CALD1^+^ macrophages from ulcerative colitis patients (Fig. 2d). Additionally, we then leveraged a monocle2 analysis approach and confirmed the origin of IL-1B^+^ macrophages among monocyte-macrophages from both Crohn’s disease and ulcerative colitis patients. In parallel, C1QB^+^ macrophages appear to be mature macrophages (Fig. 2e-f).

### Macrophages modulate intestinal and extraintestinal homeostasis during IBD

Dysregulation of intestinal immune response results in an increase in myeloid cell-derived cytokines, chemokines as well as other pro-inflammatory mediators, which further influence the fates and functions of immune cells. GM-CSF, an ILC3-derived or IL-23-stimulated mediator, has been found to contribute to a powerful payload in elevating the expression of IL-1β and IL-23 by monocytes-macrophages lineage within the context of *C. rodentium* infection or DSS-induced colitis [8, 40, 135, 140]. TNF-α and iNOS are considered to be representative colitogenic mediators derived from monocyte-macrophage lineages that contribute to the development of colitis [61, 141]. Infiltrated-monocytes and immature macrophages are also enriched of pro-inflammatory mediators including TREM1, TREM2, S100A8, S100A9, CXCL2, CXCL8, SPP1, GPNMB, AREG, HBEGF, NRG1 and SOD2, leading to the persistence of intestinal inflammation [10, 38, 130]. Evidence has been emerging that colitis-associated monocytes upregulate the expression of TREM1, which is reckoned as a potent amplifier of pro-inflammatory responses [45, 62, 142]. Consistently, TREM1 deficiency compromises pro-inflammatory features of the monocyte-macrophage lineages, thus culminating in the inhibition of colitis in mice [8]. However, intestinal mucosal inflammation also induces anti-inflammatory features of the monocyte-macrophage lineage, which is instrumental in the secretion of anti-inflammatory mediators, including IL-10, TGF-β, vascular endothelial growth factor (VEGF), and metalloproteinases with the stimulation of inhibitory mediators, thus participating in the process of wound healing and tissue repair [11, 28, 41, 43, 45, 50, 57, 78, 113, 143]. Notably, macrophage cell surface receptors, including PD-L1 and PD-L2, are involved in the suppression of inflammation and ultimately delay the injury repair [57, 78]. Nonetheless, evidence has shown that anti-inflammatory cytokines are transferred to lysosomes and degraded rather than exerted functions in the intracytoplasmic of Crohn’s disease macrophages, and this dysregulated process is triggered by *E. coli* or TLR agonists [136]. These data suggest that compromised macrophage cytokine secretion underlies acute inflammation in Crohn’s disease.

Apart from cytokine secretion, the monocyte-macrophage lineage contributes to the process of epithelial cell proliferation, angiogenesis, fibrosis, and tissue repair, while tissue repair fails due to the domination of proinflammatory macrophages and the inappropriate functions of macrophages in the inflamed intestinal mucosa [57, 144]. After stimulating with inflammatory signaling, intestinal macrophages are also preferentially endowed with a feature of wound healing by regulating the generation of WNT [109, 110], MMPs [31], PGE2 [108], VEGF [40], HGF [106], as well as the signaling pathways like NOX1 signaling activated by annexin A1 (ANXA1) [111, 112], mTORC1 − arginase-1 axis [115], CSF1R-related signaling [2, 6, 116], CREB-WISP1 axis [113], and MyD88 signaling [11, 114], which are involved in the intestinal stem cell proliferation and its differentiation into intestinal epithelial cells, Paneth cells and goblet cells, thus contributing to tissue repair of inflamed intestine. Previous data have uncovered that macrophages from Crohn’s disease patients produce less HGF than healthy controls, leading to ineffective epithelial repair [106]. Hypoxic macrophages produce WNT in a HIF-1-dependent manner, which impairs epithelial autophagy in the intestinal mucosa of IBD patients [109]. The activation of WNT signaling during TNBS-induced colitis also contributes to wound healing in a STAT6-dependent manner [110]. Macrophage-derived PGE2 activates the WNT/β-catenin signaling in intestinal stem cells and facilitates stem cell self-renewal by binding to its receptors EP1/EP4. This process can be further amplified after a combination of 5-hydroxytryptamine and its receptors HTR2A/3A in macrophages [108]. During gut inflammation, the expression of VEGF from macrophages is upregulated, leading to the activation of perivascular fibroblasts and the reduction of CXCR7 expression [40]. ANXA1 and ROS have been found to be increased in the intestinal epithelia cells and infiltrating immune cells in the inflamed mucosa of patients with ulcerative colitis, which contribute to tissue repair during intestinal inflammation in a NOX1-dependent manner [111, 112]. Moreover, intestinal microbiota orchestrates the expression of NOX1 by TLR signaling to maintain intestinal immune homeostasis [112], and intestinal macrophages can also produce polyamines to synergistically induce intestinal epithelial cell proliferation via the mTORC1 − arginase-1 axis [115]. It is noteworthy that the CSF/CSF1R signaling acts as a modulator of intestinal epithelial differentiation, except for its affection for the intestinal monocyte-macrophage maturation. Conversely, macrophage depletion through CSF1R blockage leads to the failure of intestinal epithelial cell differentiation [116]. An upregulation of macrophage-generated IL-10 in the context of intestinal inflammation activates epithelial CREB and facilitates synthesis and secretion of the pro-repair WISP-1 subsequently [113]. On the contrary, IL-10 deficiency promotes the apoptosis of intestinal epithelial cells in mice, which is reversed via the neutralization of TNF-α and iNOS [61, 141]. Additionally, evidence has emerged that Myd88 signaling pathway is indispensable in the enrichment of intestinal Ptsg2-expressing stromal cells and the activation of colonic epithelial response to inflammation during DSS-induced mouse colitis [114]. With the application of novel technologies, there is potential to further explore the underlying mechanisms whereby macrophages are involved in tissue repair and intestinal epithelial proliferation.

Accumulating lines of evidence have suggested that fibrosis contributes to the intestinal tissue repair after an inflammatory attack, but dysregulation of intestinal mucosal fibrosis leads to pathological fibrosis or even scarring [6, 8, 34, 38, 40, 57, 144]. Mechanically, macrophages are involved in the recruitment and differentiation of tissue fibroblasts into myofibroblasts and upregulate the synthesis of ECM components [57, 121] via the production of MMPs, tissue inhibitor of metalloproteinases 1 (TIMP1), PDGF, TGFβ1, IGF-1 and CTGF [57, 121, 122]. MMPs participate as enzymes for the degradation of ECM proteins or triggers for fibrosis [57]. For example, macrophage-derived MMP9 and MMP12 are markedly induced in the context of IL-13 stimulation or CCL4 and thioacetamide-induced liver fibrosis and subsequently promote fibrosis [145, 146]. The TGFβ-dependent activation of MMP2 is negatively regulated by BMP7 [147]. In contrast, macrophage-derived TIMP1 contributes to the normalization of the pro-fibrotic hematopoietic-vascular niche, and these macrophages are recruited by endothelial-produced endocrine chemokines [148]. PDGF and TGFβ1 have long been recognized as macrophage-derived growth factors that promote angiogenesis and fibrosis, thus playing an indispensable role in tissue rehabilitation [149]. *S Typhimurium* infection results in chronic colitis, as evidenced by transmural ECM deposition within the intestine and the activation of fibrotic response, concomitantly with the enhancement expression of TGFβ1 and IGF-1 [150]. CTGF, identified as a fibrotic marker, has been found to be upregulated during inflammatory diseases, which further facilitates the expression of pro-inflammatory cytokines and chemokines to participate in the fibrotic process. On the contrary, the elimination of CTGF in mice restrains the expression of pro-inflammatory mediators and fibrosis-associated biological processes [150]. Moreover, IL-36α^+^ macrophages and immature macrophages in the intestinal mucosa of IBD patients have been reported to be strongly associated with inefficient tissue wound healing and contribute to the infliction of pathological fibrosis or scarring in the intestinal mucosa, leading to chronic tissue injuries or chronic inflammation, and finally culminating with the occurrence of intestinal stricture, stenosis or obstruction [124, 151]. Through the integration of single-cell transcriptomics and spatial transcriptomics, a recent study has provided compelling evidence to show that hepatocyte growth factor activator (HGFAC) Arg509His (R509H), a risk variant for Crohn’s disease activated by thrombin protease activity, restricts fibroblast-mediated tissue reestablishment in the inflammatory intestine on account of impairing proteolytic activation of the growth factor macrophage-stimulating protein (MSP). Notably, a reduction of growth factors causes an impairment of wound-associated epithelial cell differentiation and retinoic acid (RA) generation, leading to delayed repair [152].

Evidence shows that the functions and maturation process of intestinal macrophages are consistent among animals and humans in both steady and inflammatory states, and prompts that animal models contribute to a potent payload of exploring the definite properties of macrophages in intestinal mucosa [11]. Animal models of intestinal inflammation, such as the DSS-induced colitis model, 2,4,6-trinitrobenzene sulfonic acid (TNBS)-induced colitis model and transfer of radio-labeled autologous blood monocytes into mice, have verified an influx of monocytes as well as eosinophils, and the suppression of macrophage maturation during these situation [10, 11, 43, 50]. In addition, published data have also elucidated that specific microbiota participates in the modulation of macrophage formation and functions in DSS-induced colitis. For example, the enrichment of *Enterobacteriaceae* microbiota in the inflammatory enteric cavity leads to the accumulation of circulating monocytes. Bacterial hemolysin is instrumental in persisting the activation of macrophage NOD-like receptor thermal protein domain associated protein 3 (NLRP3) inflammasomes, which mediates the excessive expression of IL-1β triggered by the pathogen *Salmonella* [40]. Synchronously, *Helicobacter hepaticus*-induced colitis enlightens the paramount role of GM-CSF in recruiting monocytes [40, 140].

**Fig. 3:**
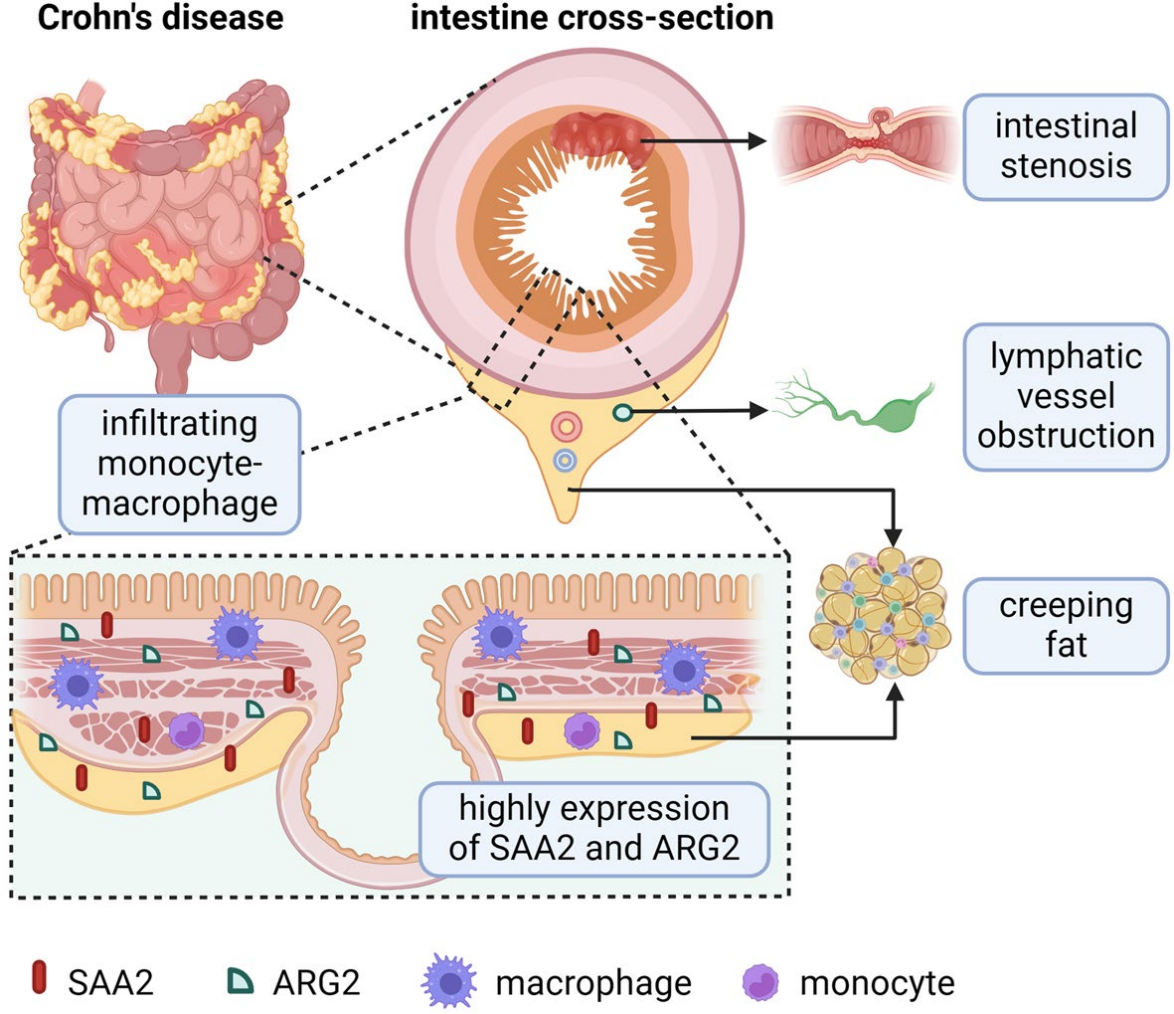
Macrophages modulate mesentery immune homeostasis in IBD patients. The development of creeping fat, mesenteric fibrosis as well as lymphatic vessel obstruction in the mesentery are reckoned as the characteristics of IBD patients. Macrophages infiltrate in the mesentery and involve in the modulation of extraintestinal immune homeostasis in IBD patients via macrophage-associated genes including SAA2 and ARG2, which are highly expressed in the mesentery and intestinal mucosa

Apart from the regulatory processes in the intestine, macrophages are also involved in the modulation of extraintestinal homeostasis in IBD patients, which is ascribed to the interaction crosstalk between the intestine and its accessory tissues, including mesenteric adipose tissue, mesentery, and mesenteric lymph nodes [153, 154]. By analyzing integrative multi-omics between proteomic and microbiome, our recent data have identified that macrophages-derived serum amyloid A2 (SAA2) is involved in the regulation of adaptive immunity in mesentery and mesenteric lymph nodes, and Th17 immunity in mesenteric adipose tissue, which is further confirmed in both serum and fecal samples as a potential diagnostic biomarker [13]. Previous studies have demonstrated that SAA2 is illustrated as a significant inducer to macrophages- or Th17 cell-mediated immunopathology [155]. Our study thus indicates that SAA2 is positively correlated with the microbial transporters ugpE and ugpC and short-chain fatty acids-producing genera, including *Dorea* and *Butyricicoccus*. In contrast, the negative correlation between SAA2 and microbial gerKC and flaG is also identified [13]. In addition, macrophage-associated gene arginase-2 (ARG2) is increased in inflamed intestinal mucosa as well as intestinal accessory tissues (i.e., mesenteric adipose tissue, mesentery, and mesenteric lymph nodes) from Crohn’s disease patients [13], and it is found to be localized at the mitochondria and upregulated by the IL-10/miR-155 axis in pro-inflammatory macrophages, thus contributing to the enhancement of oxidative phosphorylation via the suppression of HIF-1α and IL-1β in inflammatory macrophages [156]. Taken together, these conceptions open new horizons for prospects and emphasize a potential future for scrutinizing the feature of macrophages in intestinal accessory tissues from IBD patients (Fig. 3).

### Susceptibility genes are associated with the diverse functions of macrophages in IBD

In addition to the delicate immune regulations of intestinal macrophages and the underlying mechanisms as mentioned above, more susceptibility genes have gradually been discovered in IBD patients, which are ascribed to the advancement of nucleic acid sequencing technology, like GWAS and ImmunoChip [14, 16, 17, 157, 158]. Copious amounts of susceptibility genes are found to be related to the maturation and function of monocyte-macrophages lineage (Table 2). Susceptibility loci related to intestinal macrophage functions have been unraveled in Crohn’s disease patients, including NOD2, ATG16L1, CX3CR1, IL12p40, IL23R, JAK2, STAT3, and protein tyrosine phosphatase non-receptor type 2 (PTPN2) [14, 16, 17, 158–160]. NOD2 is the first conferred and one of the most important susceptibility genes of Crohn’s disease [161, 162], which participates in regulating inflammatory response to bacterial antigens [2, 6, 159, 160, 163]. NOD2 recognizes the bacterial-derived muramyl dipeptide by suppressing TLR2-mediated activation of NF-ĸB [164], and the mutation of NOD2 results in the enhancement of TLR2-driven activation of NF-ĸB [165]. By using the DSS-induced colitis model in NOD2-deficient zebrafish, the deficiency of NOD2 is observed to contribute to an impairment in the monocyte-macrophage lineage, while the mutation of NOD2 leads to the overexpression of collagen, all of which are associated with pathological fibrosis [6]. Together with ATG16L1, another susceptibility locus for Crohn’s disease [16, 157], NOD2 triggers the process of autophagy by accumulating at the site where bacteria gather [14, 160, 166, 167]. Autophagy-related mediators, including RIPK2, ATG5, and ATG7 participate in this process [160, 168, 169]. Impaired antifungal responses due to the missense mutation of another IBD-related gene CX3CR1 in myeloid cells lead to an increase in intestinal and extraintestinal inflammatory diseases [2, 170]. Several components of the IL-23 signaling pathway are also confirmed as IBD susceptibility genes, including IL23R, IL12B, STAT3, and JAK2, which help to explain the regulatory effect of these mediators on intestinal mucosal immune homeostasis in IBD patients [160, 167, 171–173]. IL-23 is derived from macrophages and DCs, and induces the expression of IL-17 from T cells, which participates in the regulation of intestinal immune homeostasis [174]. In addition, the JAK2-STAT3 signaling is involved in the modulation of macrophage proliferation [14, 175]. More studies, assisted by scRNA-seq analysis, have revealed the enhancement of proliferation and glycolytic metabolism of inflammatory macrophages in individuals with JAK2 mutation, resulting in the activation of AIM2 inflammasome and the aggravation of inflammation [175]. On the other hand, PTPN2 has been observed as a susceptibility gene to ulcerative colitis [176, 177], which participates in the regulation of the JAK2-STAT3 signaling, modulation of IL-2 responsiveness, and maintenance of intestinal epithelial barrier integrity [176, 178].

**Table 2:** IBD-related susceptibility genes are associated with macrophage functions

Gene name	Functions
NOD2	1) Regulate inflammatory response to bacterial antigens [2, 6, 159, 160, 163]
	2) Recognize bacterial-derived muramyl dipeptide [164]
	3) Suppress TLR2-mediated activation of NF-^-^B [164, 165]
	4) Maintain the homeostasis of monocyte-macrophage lineage [6]
	5) Regulate collagen production and fibrosis [6]
	6) Trigger autophagy process [160, 166, 167]
ATG16L1	Trigger autophagy process [160, 167]
CX3CR1	Involve in invading pathogen process and antifungal responses [2, 170]
JAK2	1) Modulate macrophage proliferation [14, 175]
	2) Induce T cell-mediated IL-17 [174]
STAT3	1) Modulate macrophage proliferation [14, 175]
	2) Induce T cell-mediated IL-17 [174]
IL23R	Induce T cell-mediated IL-17 [174]
PTPN2	1) Regulate the JAK2-STAT3 signaling [176]
	2) Maintain the integrity of intestinal epithelium [176]
	3) Modulate IL-2 responsiveness [178]
LILRB3	Enhance M2-like macrophage polarization [179]
IL21R	Amplify macrophage activation [180]
GTF2I	Modulate M2-like macrophage polarization [181]
RUNX3	1) Maintain of the expression of CD4 and CD14 in the monocyte-macrophage lineage [182]
	2) Orchestrate macrophage maturation [182]

*NOD2* nucleotide binding oligomerization domain containing 2, *ATG16L1* autophagy related 16 like 1, *CX3CR1* C-X3-C motif chemokine receptor 1, *JAK2* Janus kinase 2, *STAT3* signal transducer and activator of transcription 3, *IL23R* interleukin 23 receptor, *PTPN2* protein tyrosine phosphatase non-receptor type 2, *LILRB3* leukocyte immunoglobulin like receptor B3, *IL21R* interleukin 21 receptor, *GTF2I* general transcription factor Iii, *RUNX3* RUNX family transcription factor 3

Recently, we have verified 54 new IBD-associated genetic loci in East Asian ancestry, like RUNX3, ADAP1, IL21R, GTF2I, and LILRB3 [17]. Notably, deficiency of LILRB3 facilitates macrophage polarization into an pro-inflammatory M1-like phenotype, contributing to the worsening of intestinal inflammation [179]. IL21R has been identified to promote macrophage activation, participate in pathogen-induced Th2 responses, and play a paramount role in inflammation and chronic fibrotic diseases [180]. The mutation of GTF2I fails to the induction of anti-inflammatory M2-like polarization in macrophages [181]. RUNX3 is a CXCL12-dependent transcription factor that is involved in the maintenance of the expression of CD4 and CD14 in the monocyte-macrophage lineage, which orchestrates the maturation of macrophages in the intestine [182].

### Macrophage-directed therapeutics for IBD

Given the paramount role and heterogeneity of macrophages in the development of IBD [130], we therefore interrogate the macrophage-directed IBD therapeutics. Based on the mechanisms underlying immunoregulation of macrophages on intestinal immune homeostasis, therapeutic approaches focus on modulating processes including phagocytosis, bactericidal effect, secretion of mediators, involvement in tissue repair and fibrosis, and the maturation of macrophage [2]. Classical drugs for treating IBD directly inhibit inflammation of macrophages in the intestinal mucosa. Corticosteroids suppress the activation of NF-ĸB and activator protein 1 (AP-1), thus contributing to monocyte maturation to macrophage [183, 184]. Mutually, the activation of NF-ĸB is also downregulated by 5-aminosalicylate [185]. In parallel, methotrexate is reckoned as an inhibitor of thymidylate synthase, which involves pro-inflammatory cytokine expression from macrophages [186]. In addition, azathioprine and 6-mercaptopurine are involved in the reduction of JUN N-terminal kinase (JNK) phosphorylation, which is ascribed to the metabolized 6-thioguanine triphosphates-dependent Ras-related C3 botulinum toxin substrate 1 (Rac1) activity [187]. With the increasing application scenarios and diversified types of biologics, the immunomodulatory effects of biological therapies have been investigated in IBD. Evidence has demonstrated that administration of anti-TNF mAb (e.g., infliximab) could facilitate maturation of CD68^+^CD206^+^ macrophages in the intestinal mucosa of IBD patients and concomitantly inhibiting T cell proliferation [6, 188]. Moreover, previous studies have also elucidated that another anti-TNF mAb (e.g., adalimumab) enables to constrain immature macrophage (CD14^+^HLADR^int^) infiltration in inflamed intestine of Crohn’s disease patients [6, 189]. Since the JAK2-STAT3 signaling is involved in the modulation of macrophage maturation and proliferation [14, 175], a target therapeutic approach using JAK inhibitors has become a hot topic worldwide. Tofacitinib, as a JAK 1/3 inhibitor, appears to suppress pro-inflammatory M1 macrophages and promotes anti-inflammatory M2 macrophages and M2-associated markers, thus contributing to maintaining intestinal barrier function through regulating the expression of tight junction protein, constraining the JAK2-STAT3 signaling, and downregulating the secretion of IL-6 and IL-22 [176, 190]. Taken together, therapeutic strategies through suppressing monocyte infiltration, downregulating the expression or the activation of pro-inflammation cytokines, bolster the maturation and M2-like polarization of macrophages, and upregulating the secretion of anti-inflammation cytokines may contribute to the resolution of intestinal inflammation in IBD.

## Conclusion

Emerging evidence has highlighted the notion that macrophages possess a striking degree of plasticity, heterogeneity, and adaptation, which makes it necessary to define macrophage subpopulations in the intestine according to their features and functions. Importantly, these data could provide benefits for a precise directional effect on translational study, diagnosis, and precision medicine in IBD [2, 35, 47, 67, 90]. Except for intestinal macrophages, extraintestinal monocyte-macrophages also gain attention from recent studies, especially mesenteric monocyte-macrophages in IBD [13, 153, 154]. SAA2 and ARG2 have been observed to upregulate in inflamed intestinal mucosa and intestinal accessory tissues, being associated with immunoregulation on intestinal macrophages [13]. Given the plasticity and heterogeneity of intestinal macrophages, as well as the diverse functions performed by extraintestinal monocyte-macrophages, which may also influence the immune homeostasis in IBD, the simplex paradigm has been challenged, improved, and enlarged. To better illustrate the precise molecular interactions between macrophages and other immune cells and dissect the potential immunoregulation of the newly identified susceptibility genes on macrophages in intestinal mucosa, cell–cell crosstalk and niche-specific functions of intestinal macrophages have gained a diverse array of attention, and an in-depth investigation on the underlying mechanisms is warranted [7, 8, 13, 29].

Decades ago, studies on IBD patients were limited by the acquisition of samples and the reconstruction of the intestinal microenvironment. It is encouraging and increasingly apparent that tremendous strides made in research of intestinal macrophage biology are a consequence of novel technology advances, including scRNA-seq [10, 29, 128], spatial transcriptomics (ST) [191–193], CosMx Spatial Molecular Imaging [130], seq-scope [194], GeoMx Digital Spatial Profiler (DSP) [195, 196], spatially-resolved transcript amplicon readout mapping (STARmap) [197], spatially enhanced resolution omics-sequencing (Stereo-seq) [198], and omics technologies [13]. The development of novel technologies responsive to the unmet need of researches will help us to understand better the pathogenesis of IBD, the relationship between macrophages and the intestinal accessory tissues in IBD, and the features of the newly founded susceptibility genes of IBD, which is foreseeable to get rid of limitations and emphasize prospects for potential therapeutic intervention.

## Data Availability

No datasets were generated or analysed during the current study.

## Abbreviations

AGM: Aorta-gonad-mesonephros
ARG2: Arginase-2
AP-1: Activator protein 1
CSF1: Colony-stimulating factor 1
CSF1R: Colony-stimulating factor 1 receptor
CTGF: Connective tissue growth factor
DSP: Digital Spatial Profiler
DSS: Dextran Sulfate Sodium Salt
ECM: Extracellular matrix
EMP: Erythro-myeloid precursors
GF: Germ-free
GWAS: Genome-wide association studies
GM-CSF: Granulocyte–macrophage colony stimulating factor
HGF: Hepatocyte growth factor
HSCs: Hematopoietic stem cells
IBD: Inflammatory bowel disease
IGF-1: Insulin-like growth factor 1
JNK: JUN N-terminal kinase
LP: Lamina propria
METs: Macrophage extracellular traps
MHC II: Major histocompatibility complex II
MMPs: Matrix metalloproteinases
NLRP3: NOD-like receptor thermal protein domain associated protein 3
OXPHOS: Oxidative phosphorylation
PDGF: Platelet-derived growth factor
PGE2: Prostaglandin E2
Rac1: Ras-related C3 botulinum toxin substrate 1
ROS: Reactive oxygen species
SAA2: Serum amyloid A2
scRNA-seq: Single-cell RNA-sequencing
ST: Spatial transcriptomics
STARmap: Spatially-resolved transcript amplicon readout mapping
Stereo-seq: Spatial enhanced resolution omics-sequencing
TNBS: 2,4,6-Trinitrobenzene sulfonic acid
TREM1: Triggering receptor expressed on myeloid cells 1
TS: Thymidylate synthase
YS: Yolk sac
ZEB2: Zinc finger E-box-binding homeobox 2
